# Pyoderma gangrenosum in a patient with chronic granulomatous disease: A case report

**DOI:** 10.1097/MD.0000000000007718

**Published:** 2017-08-04

**Authors:** Sideris Nanoudis, Afroditi Tsona, Olga Tsachouridou, Petros Morfesis, Georgia Loli, Adamantini Georgiou, Pantelis Zebekakis, Symeon Metallidis

**Affiliations:** 1st Department of Internal Medicine, AHEPA University Hospital, Aristotle University of Thessaloniki, Thessaloniki, Greece.

**Keywords:** Pyoderma gangrenosum, Chronic granulomatous disease, Primary immunodeficiency

## Abstract

**Rationale:**

The simultaneous occurrence of pyoderma gangrenosum (PG) and chronic granulomatous disease (CGD) is uncommon and few cases have been reported worldwide.

**Patient concerns:**

PG is a rare, chronic, ulcerative, neutrophilic skin disease of unknown etiology that requires immunosuppressive treatment. CGD belongs to Primary Immune Deficiencies in which the main defect lies in an inability of the phagocytic cells to generate superoxide making patients susceptible to serious, potentially life-threatening bacterial and fungal infections.

**Diagnoses:**

In this manuscript, we present a case of ulcerative pyoderma gangrenosum in a 28-year-old man with recent diagnosis of chronic granulomatous disease during hospitalization for resistant pulmonary tuberculosis complicated with *Aspergillus* infection.

**Interventions:**

Second-line therapy with dapsone and intravenous immunoglobulin was initially administered but eventually corticosteroids were added to treatment because of disease progression and further ulceration.

**Outcomes:**

Patient's ulcers were gradually healed with no side effects.

**Lessons:**

Corticosteroids could be used under close monitoring for the treatment of PG in a patient with CGD, despite the increased risk for infections.

## 1 Introduction

Pyoderma gangrenosum (PG) is an idiopathic, ulcerative, neutrophilic, non-infective chronic inflammatory dermatitis of low frequency (10 per 1,000,000 individuals) that requires corticosteroids and immunosuppressive treatment.^[1–3]^ On the other hand, chronic granulomatous disease (CGD) is a rare (8 per 1,000,000 individuals), inherited disorder of the immune system characterized by an inability of the phagocytic cells (neutrophils, monocytes, and macrophages) to produce superoxides. As a result, these patients suffer from recurrent infections, often of unusual pathogens or of unusual severity and frequency and should take lifelong antibacterial and antifungal prophylaxis.^[4–6]^ The simultaneous coexistence of both diseases has been reported only a few times in literature^[7–10]^ and, furthermore, imposes cautious management of PG, due to the underlying pathophysiology.

## 2 Patient information

**Figure 1:**
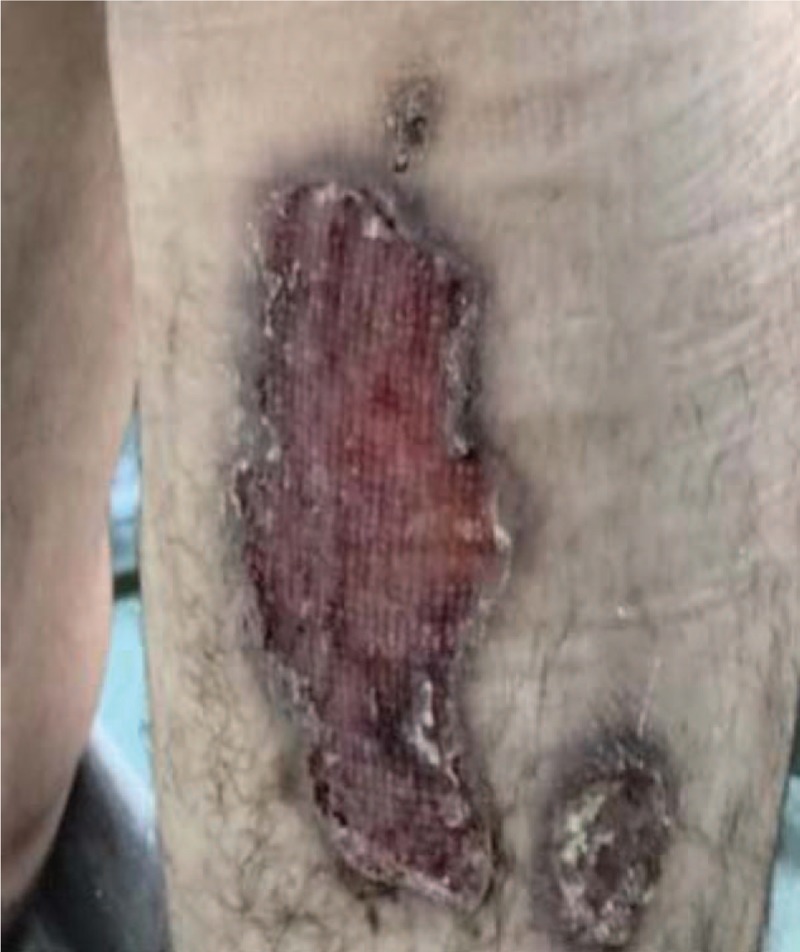
Pyoderma gangrenosum on the right thigh (12 months old).

**Figure 2:**
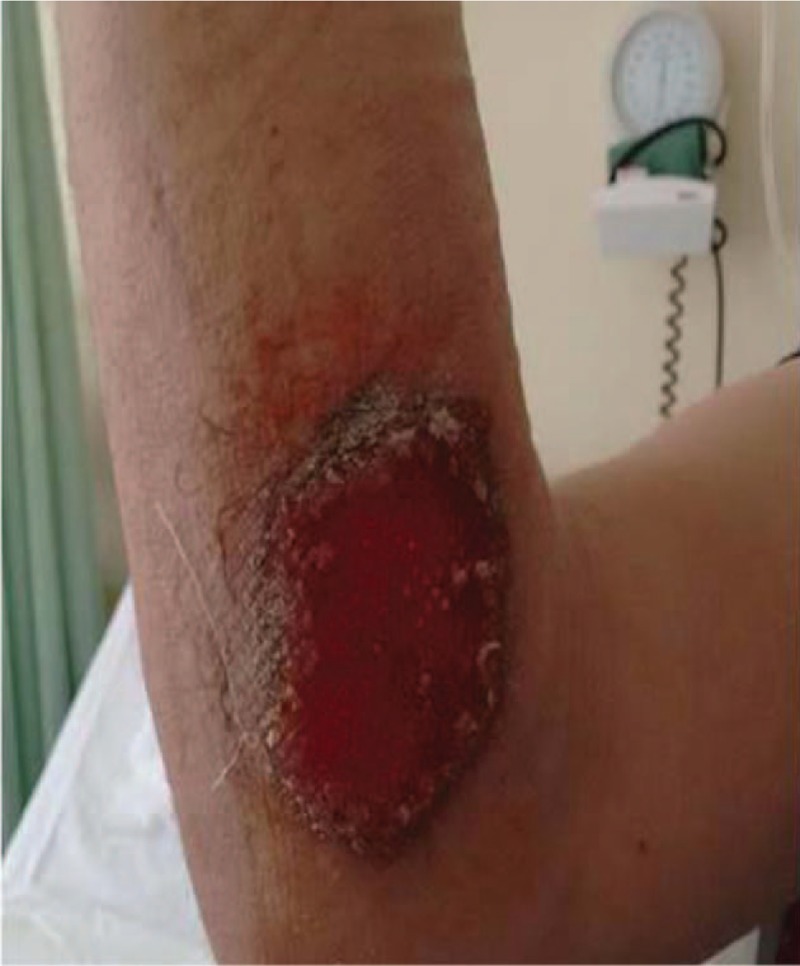
Pyoderma gangrenosum on the right forearm (3 months old).

**Figure 3:**
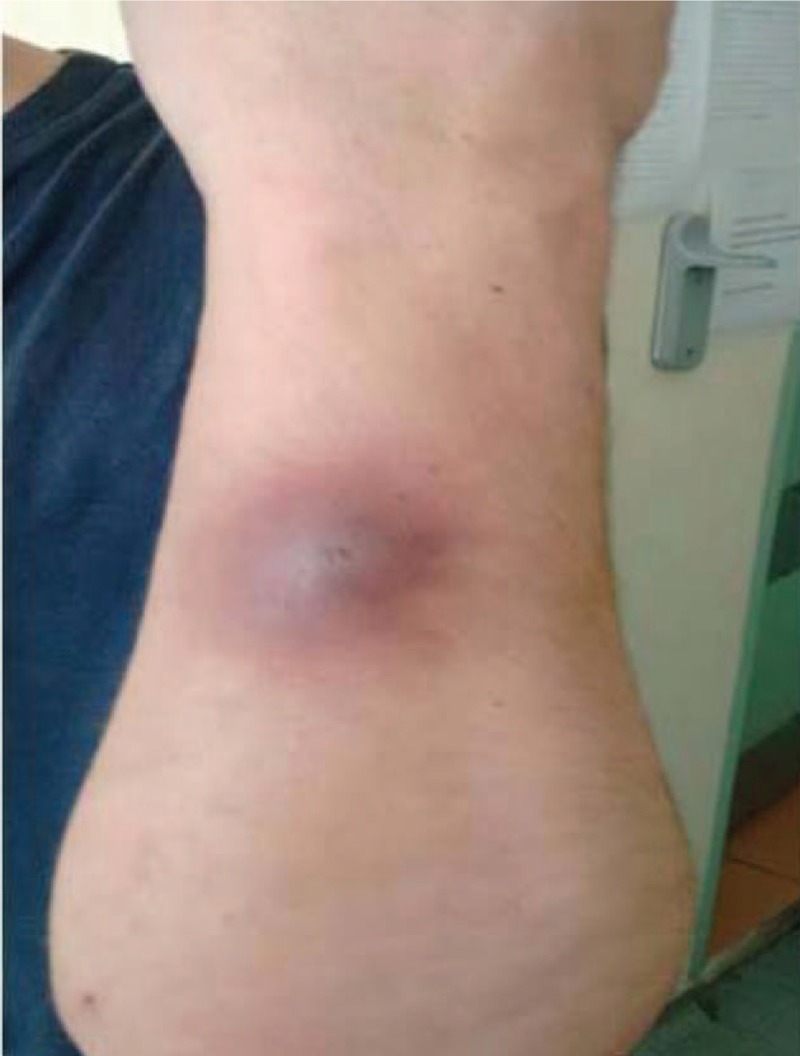
Cutaneous lesion, resembling a furuncle, on the left forearm appeared a week ago.

A 28-year-old Caucasian man, former intravenous drug user, was admitted to our Internal Medicine Department, because of 2 ulcerative cutaneous lesions (indicative of pyoderma gangrenosum), 1 on the right thigh (about a year old) and a 2nd one on the right forearm (3 months old) (Figs. 1 and 2). A 3rd lesion was also present on the left forearm resembling to furuncle erupted a week ago (Fig. 3). The patient was diagnosed with CGD only 2 years ago, during a hospitalization for resistant, miliary tuberculosis complicated with *Aspergillus* pulmonary infection. Dihydrorhodamine (DHR) flow cytometry was initially used to detect reduced superoxide production of stimulated neutrophils, followed by molecular mutation analysis which revealed a defect in p47^phox^ component of nicotinamide adenine dinucleotide phosphate (NADPH) oxidase. He received a total of 18 months of antituberculosis treatment consisted of 7 agents (isoniazid, rifampicin, ethambutol, pyrazinamide, levofloxacin, amikacin, and cycloserine) which was completed 3 months before his first admission to our department. Bacillus Calmette Guerin (BCG) vaccine was administered at the age of 6, but no evidence of local or disseminated complications indicative of BCG infection due to the underlying CGD were mentioned.^[11–13]^ His rest medical history consists of recurrent infections throughout childhood, adolescence, and adulthood including meningitis at the age of 9, respiratory and urinary tract infections, Staphylococcus skin infections and inactive chronic hepatitis C with undetectable HCV viral load (VL below 15 IU/mL). Conversely, there was no family history of CGD and patient's parents as well as his sister did not suffer from severe or frequent infections.

### 2.1 Clinical findings

Physical examination revealed no pathological signs. The patient, apart from the cutaneous lesions, was asymptomatic and afebrile. Interestingly, a pathergy sign was positive on the right thigh as satellites lesions proximal to the primary skin lesion appeared after accidental injury. His blood pressure was 120/75 mm Hg and pulse rate 75 beats per minute. Oxygen saturation was also normal.

### 2.2 Diagnostic assessment

**Figure 4:**
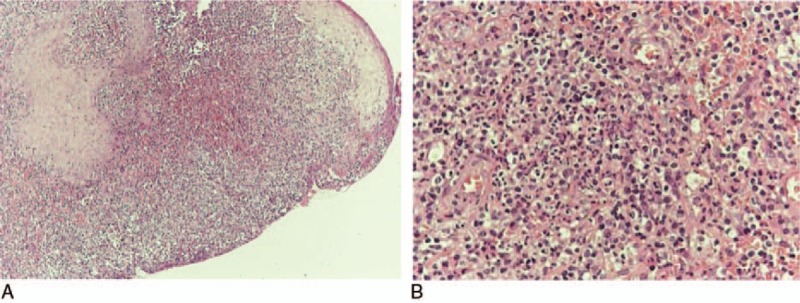
(A, B): Skin biopsy presenting epidermal ulceration with exudation and inflammatory granulomatous tissue (A), having areas with central abscess formation, surrounded by granulomatous inflammation (B) (A: HE ×100, B: HE ×400).

The laboratory tests revealed a moderate microcytic, hypochromic anemia (Hb 9.5 g/dL), C-reactive protein (CRP) level of 4 mg/dL (normal range 0–0.8 mg/dL), and erythrocyte sedimentation rate (ESR) level of 50 mm/h (normal range <15 mm/h). On the other hand, rheumatoid factor (RF), antinuclear antibody (ANA), antineutrophilic cytoplasmic antibody (ANCA), antiphospholipid antibody, and test for cryoglobulins were all negative. Triplex ultrasound revealed no venous or arterial thrombosis while computed tomography (CT) of thorax and abdomen confirmed merely slight hepatosplenomegaly. Ulcer cultures derived from skin biopsy were negative for common bacteria, fungus, Mycobacterium tuberculosis, and atypical mycobacterium. Moreover, histological findings were indicative of pyoderma gangrenosum characterized by epidermal ulceration and granulomatous inflammation consisting of neutrophils, lymphocytes, and plasma cells, and, also, excluded vasculitis and cancer. In addition, Periodic acid–Schiff (PAS) stain did not show any evidence of fungal infection (Fig. 4).

### 2.3 Therapeutic intervention

**Figure 5:**
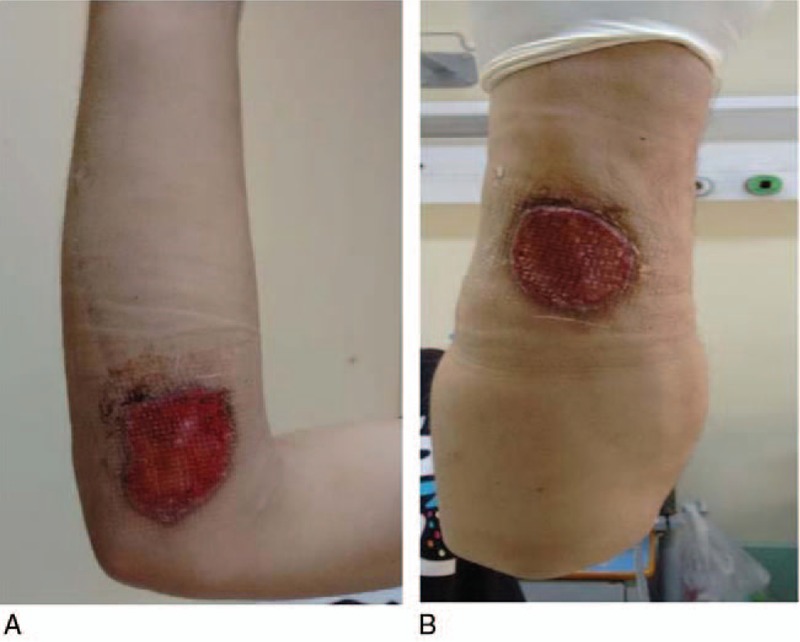
(A, B): After 1 month of treatment with dapsone and intravenous immunoglobulin no improvement was noticed on the right forearm (A) and the lesion on the left forearm (B), also, progressed to ulcerative pyoderma gangrenosum.

High dose corticosteroids are usually the first line systemic therapy for PG. Cyclosporine A is added as a steroid sparing agent or because of incomplete response to corticosteroids.^[14–16]^ However, in our case, due to the underlying CGD and the recent medical history of miliary tuberculosis accompanied with *Aspergillus* infection it was decided to administer a second line combination therapy with dapsone 100 mg twice a day and 6 courses of 2-day administration of intravenous immunoglobulin (IVIG) of 1 g/kg/d monthly, along with local wound care. Itraconazole 300 mg per day and Sulfamethoxazole/Trimethoprim 800/160 mg once a day were also added as a prophylaxis for the CGD. One month later, not only no improvement was marked, but also the lesion on the left forearm had progressed to a third ulcer (Fig. 5A and B). Upon being informed of the potential risks of his decision, the patient consented to escalate his therapy by adding corticosteroids with close clinical and laboratory observation. Initially, intravenous pulse methylprednisolone 1 g/d was administered for 5 days and after that, oral methylprednisolone 16 mg twice a day which was tapered 4 mg every 4 days. The remaining dose of methylprednisolone was 4 mg every 2 days while simultaneous treatment with dapsone 100 mg twice a day and 2-day IVIG 1 g/kg/d monthly was continued.

### 2.4 Follow-up and outcomes

**Figure 6:**
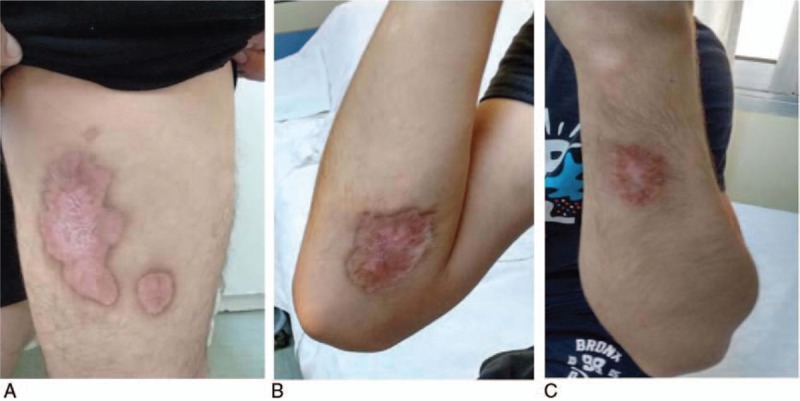
(A, B, C): All 3 lesions were healed 3 months after adding methylprednisolone to treatment.

The patient's condition gradually improved, with the lesions being healed over time 3 months after the administration of methylprednisolone (Fig. 6A, B, C). Blood tests were also improved with increase of hemoglobulin and restoration of inflammatory markers (Hb 11.7 g/dL, CRP 0.1 mg/dL, and ESR 5 mm/h) while the patient had no sign of infection or other side effect from the corticosteroid treatment.

## 3 Discussion

Pyoderma gangrenosum is a chronic, ulcerative, non-infective, neutrophilic skin disorder of unknown etiology.^[1–3]^ Cutaneous lesions usually begin as pustule or vesiculopustule and progress to an ulcer or deep erosion^[2,22]^ (in our patient, however, they began as a furuncle). Apart from the classic ulcerative PG, there are, also, other clinical variants, such as pustular PG, bullous PG, vegetative PG, and peristomal PG.^[1–2,14,16,17]^ Diagnosis is mainly clinical and derives from exclusion of other causes of cutaneous ulceration.^[1–3,14]^ On the other hand, cutaneous manifestations of CGD, apart from skin infections, include discoid lupus erythematosus-like lesions, photosensitive rashes, and aphthous ulceration (they are, also, common in X-linked female carriers) as well as seborrheic dermatitis, vesicular lesions, and facial granulomata.^[4,6,18–21]^ In our case, however, there was a higher suspicion of PG, but due to the underlying CGD, a skin infection had to be excluded. Cultures from the lesions and all the microbiological and blood tests were negative for infection, while laboratory screening for vasculitis, thrombophilic states, and vascular insufficiency were, also, negative. Finally, the biopsy was indicative of pyoderma gangrenosum and excluded vasculitis and cancer. PG is associated with systemic illnesses in half of the cases and the most common are inflammatory bowel disease, rheumatoid arthritis, systemic lupus erythematosus, hematologic diseases, and solid tumors, all of which were excluded in our patient during the diagnosis process.^[2,3,15]^

Both differences and similarities in the pathophysiology of those two diseases are extremely interesting, since as already mentioned PG is a neutrophilic dermatitis while, on the contrary, CGD's main defect lies in phagocytic cells (neutrophils, monocytes, and macrophages).^[2,4]^ More precisely, neutrophils from CGD patients fail to exhibit a respiratory burst and produce superoxides due to the absence of 1 of the components of NADPH oxidase,^[4–6]^ which in our patient was p47^phox^. Affected individuals are, therefore, susceptible to severe, potentially life-threatening bacterial and fungal infections (especially from *Staphylococcus aureus* and *Aspergillus* spp).^[4–6,23–25]^ Nevertheless, apart from the inability to produce superoxides, phagocytic cells maintain other functions, like activation from cytokines, chemotaxis, and the ability to infiltrate tissues as well as normal formation of other hydrolytic enzymes in the phagosome.^[26–29]^ This fact could explain the coexistence of both diseases in our patient. On the other hand, pathophysiology of PG is poorly understood but neutrophilic dysfunction seems to play significant role, including altered neutrophil chemotaxis and defects in phagocytosis and host defense against bacterial infections.^[30,31]^ Furthermore, abnormal leucocyte trafficking and disorganized NADPH oscillations with numerous frequencies are described in neutrophils from patients with PG, compared with sinusoidal metabolic oscillations of healthy individuals, increasing neutrophilic infiltration.^[32]^ However, it is ambiguous whether the neutrophilic dysfunction in PG is primary or secondary to other causes, such as inflammasome activation and overexpression of proinflammatory mediators, such as TNF-a, IL-1b, and IL-8.^[30]^

Mutations in p47^phox^ component of NADPH is an autosomal recessive inherited form of CGD and accounts for 25% of all cases.^[4–6]^ Autosomal recessive inherited CGD is considered to have better prognosis and is related with milder infections compared with X-linked gp91^phox^ deficient form (65% of all cases),^[4–6]^ however, in our case, recurrent infections since childhood, including meningitis, staphylococcus skin infections, and resistant pulmonary tuberculosis complicated with *Aspergillus fumigatous* coinfection were preceded. Noticeably, mycobacterium tuberculosis is not among the typical pathogens of CGD, but even so, patient not only was infected but also suffered from the resistant, military form.

Treating pyoderma gangrenosum in a patient with CGD remains a challenge. These patients must take lifelong antibacterial and antifungal prophylaxis with cotrimoxazole and itraconazole and, sometimes, treatment with interferon-gamma is also required to avoid recurrent infections.^[4–6]^ The only successful and permanent treatment that can cure the underlying genetic defect in CGD is hematopoietic stem cell transplantation and maybe gene therapy in the future.^[4–6,33,34]^ On the other hand, although there are no specific guidelines for the treatment of PG due to lack of large randomized controlled trials, most authors recommend high doses of corticosteroids for a long time (plus cyclosporine A when required) as first line therapy in PG.^[1–2,16–17]^ Dapsone, IVIG, and other immunosuppressants, including azathioprine, mycofenolate mofetil, chlorambucil, and infliximab could be used as alternative steroid-sparing agents or in presence of steroid related side effects.^[1–2,14–17]^ In addition, dapsone is considered by some authors as drug of first choice, however, in aggressive forms of PG dapsone should not be used as monotherapy but as an adjuvant agent to oral corticosteroids.^[14,17,35,36]^ Corticosteroids, however, although increasing the risk of infection, may be useful and necessary in CGD patients in case of complications due to chronic granulomatous inflammation, which is also a characteristic of this syndrome, such as colitis, gastric outlet obstruction, pericarditis, and urethral stricture.^[4–6]^ Indeed, in fulminant mulch pneumonitis steroids are mandatory and should be administered as soon as possible along with antifungal treatment.^[4,6]^ This excessive inflammatory process may represent an overwhelming response to a resolved past infection by normal cells of the immune system (such as T and B lymphocytes), and defects in phagocytosis along with increased cytokine responses due to poor superoxidegeneration.^[5,37,38]^ Nevertheless, steroids should always be administered under close observation and sometimes, indeed, specialist's opinion may be useful, especially where there is suspicion of coexisting infection or history of fungal infection.^[4]^

Apart from CGD, PG is also infrequently associated with other congenital or acquired immune deficiencies, demonstrating that dysregulation of immune system might play an important role in the pathophysiology of this disease.^[2,14]^ PG has been reported in patients with common variable primary immunodeficiency,^[39,40]^ Bruton's X-linked agammaglobulinemia,^[41,42]^ C7 deficiency,^[43]^ leukocyte adhesion deficiency type 1,^[44,45]^ as well as in HIV-positive individuals.^[46,47]^ However, the simultaneous occurrence of PG and CGD is very rare worldwide and, to the best of our knowledge, there are only a few case reports in the literature.^[7–10]^ In one of these references features of hyper-IgE syndrome were, also, present^[7]^ while others were related to peristomal PG^[8,10]^ (see, Table 1). Immunosupressants, including corticosteroids were used with caution to treat these patients and, eventually, the lesions were healed through time with no side effects.^[7–9]^ In our patient, however, it was initially decided to administer a more conservative second-line therapy with dapsone and intravenous immunoglobulin, under the fear of a severe infection (considering, also, the recent *A. fumigatus* infection) or relapse of the previous tuberculosis if corticosteroids were used.^[2,3,14,16,17]^ Patient was informed that dapsone has a slow onset of action. However, a month later and after the onset of a third ulcer as a progression of the existing furuncle on the left forearm, the patient himself asked for escalation of the treatment with steroids, understanding the potential risks of his decision. Methylprednisolone was added to treatment along with daily prophylaxis with cotrimoxazole and itraconazole. Dermatologist advice was requested for the dosage of methylprednisolone and decided to start with a 5-day intravenous pulse of 1 g/d and then proceed with a moderate dosage of about 0.5 mg/kg (16 mg twice a day) tapering 4 mg every 4 days, till the remaining dosage of 4 mg every 2 days. Systemic therapy with oral prednisone in substantial dosages of 0.5 to 1 mg/kg/d and sometimes up to 120 mg daily is usually the agent of choice in the treatment of PG.^[2,3,14,16,17]^ Nevertheless, the highest dosage of initial intravenous pulse steroid treatment was preferred (many authors recommend methylprednisone 1 g daily for 1–5 days),^[1,14,16,17]^ in order to have the advance of proceeding with lower doses of oral methylprednizolone (0.5 mg/kg/d) and taper down faster at the maintenance dose, aiming at fewer side effects. The lesions were gradually healed about 3 months later, fortunately with no adverse events and no signs of infection. Finally, the blood test results also improved, except for a remaining, mild, microcytic, hypochromic anemia that is often detected in CGD.^[4]^

**Table 1:**
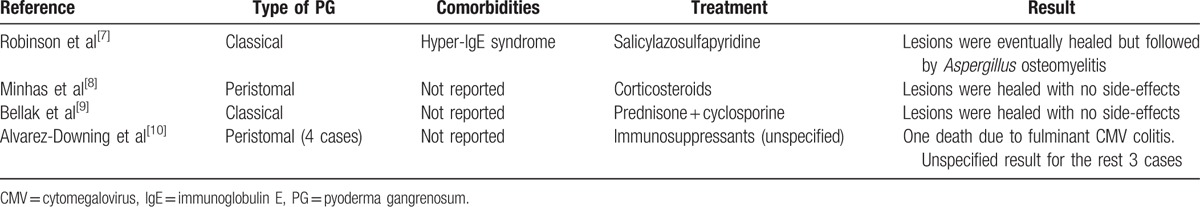
Previous clinical presentations of pyoderma gangrenosum in patients with chronic granulomatous disease.

## 4 Conclusion

In conclusion, here we report a case of pyoderma gangrenosum in a patient with a known history of chronic granulomatous disease, highlighting the coexistence of these both rare conditions. In addition, the suggested treatment of PG with corticosteroids and immunosupressants could potentially be harmful to a patient with CGD. This case report shows how combined therapy, including corticosteroids at proper dosage and close clinical and laboratory monitoring, can be applied to successfully treat pyoderma gangrenosum, overcoming the increased risk of infections.
